# Predictors of Postoperative Seizure Outcome in Low Grade Glioma: From Volumetric Analysis to Molecular Stratification

**DOI:** 10.3390/cancers12020397

**Published:** 2020-02-08

**Authors:** Tamara Ius, Giada Pauletto, Barbara Tomasino, Marta Maieron, Riccardo Budai, Miriam Isola, Daniela Cesselli, Christian Lettieri, Miran Skrap

**Affiliations:** 1 Neurosurgery Unit, Department of Neuroscience, Santa Maria della Misericordia University Hospital, 33100 Udine, Italy; 2 Neurology Unit, Department of Neuroscience, Santa Maria della Misericordia University Hospital, 33100 Udine, Italy; giada.pauletto@asuiud.sanita.fvg.it (G.P.); riccardo.budai@asuiud.sanita.fvg.it (R.B.); christian.lettieri@asuiud.sanita.fvg.it (C.L.); 3 Scientific Institute, IRCCS E. Medea, San Vito al Tagliamento, 33078 Pordenone, Italy; 4 Medical Physics, Santa Maria della Misericordia University Hospital, 33100 Udine, Italy; marta.maieron@asuiud.sanita.fvg.it; 5 Department of Medicine, Santa Maria della Misericordia University Hospital, 33100 Udine, Italy; miriam.isola@uniud.it; 6 Institute of Pathology, Santa Maria della Misericordia University Hospital, 33100 Udine Post, Italy; daniela.cesselli@uniud.it

**Keywords:** low grade glioma, seizure outcome, molecular markers, extent of resection, tumor growth pattern, ROC curves

## Abstract

The importance of the extent of resection (EOR) has been widely demonstrated as the main predictor for survival, nevertheless its effect on tumor related epilepsy is less investigated. A total of 155 patients were enrolled after a first-line surgery for supratentorial Diffuse Low Grade Gliomas (DLGGs). Postoperative seizure outcome was analyzed stratifying the results by tumor volumetric data and molecular markers according to 2016 WHO classification. Receiver operating characteristic (ROC) curves were computed to asses EOR, residual tumor volume, and ΔT2T1 MRI index (expressing the tumor growing pattern) corresponding to optimal seizure outcome. A total of 70.97% of patients were seizure-free 18 months after surgery. Better seizure outcome was observed in IDH1/2 mutated and 1p/19q codeleted subgroup. At multivariate analysis, age (*p* = 0.014), EOR (*p* = 0.030), ΔT2T1 MRI index (*p* = 0.016) resulted as independent predictors of postoperative seizure control. Optimal parameters to improve postoperative seizure outcome were EOR ≥ 85%, ΔT2T1 MRI index ≤ 18 cm^3^, residual tumor volume ≤ 15 cm^3^. This study confirms the role of EOR and tumor growing pattern on postoperative seizure outcome independently from the molecular class. Higher ΔT2T1 MRI index, representing the infiltrative component of the tumor, is associated with worse seizure outcome and strengthens the evidence of common pathogenic mechanisms underlying tumor growth and postoperative seizure outcome.

## 1. Introduction

Seizure is the most common onset symptom in patients with supratentorial diffuse low grade gliomas (DLGG), with a seizure frequency ranging from 60% to 90% [1,2]. Tumor-related epilepsy tends to manifest with focal and focal-to-bilateral tonic-clonic seizures, and more than 50% of cases show pharmaco-resistance, which contributes negatively on quality of life [3,4,5]. Recent studies have pointed out that epileptogenesis and tumor growth in DLGG may share common pathogenic mechanisms that can influence each other, thus representing two aspects of the same disease [6]. In this context, several genetic alterations have been identified as risk factors of glioma-related epilepsy. Mutations of the gene encoding the isocitrate-dehydrogenase1 (IDH1) and 2 (IDH2) can be found in about 70%–80% of DLGG [7]. These mutations have been associated with metabolic changes that are potentially epileptogenic, in accordance with the capability of IDH-mutated glioma cells to penetrate and surround the neurons in the gray matter [8,9].

Seizure outcome represents an important challenge in the daily management of DLGG patients. In particular, decision-making still varies across surgical centers given the lack of well-established and universally recognized predictors of seizure outcomes.

In the last decades, numerous studies, based on the objective evaluation of the extent of resection (EOR) has been published, demonstrating that an extensive surgery leads to increased overall patient survival and decreased malignant progression [10,11,12,13,14]. 

Although EOR has also been shown to be one of the main strongest significant predictor markers for seizure outcome [7,15,16,17,18], its predictive role has not been completely clarified in complex predictor models for epileptic outcome stratification combining molecular and tumor volumetric data.

We assessed the capability of the main clinical, molecular, and radiological data used in DLGG, including the tumor growing pattern and EOR, to predict postoperative seizure outcome, with the aim to provide useful tools for the early identification of postoperative seizure persistence and for the refinement of medical treatment tailoring in subjects with refractory epilepsy.

## 2. Results

### 2.1. Study Population and Postoperative Seizure Outcome

**Figure 1:**
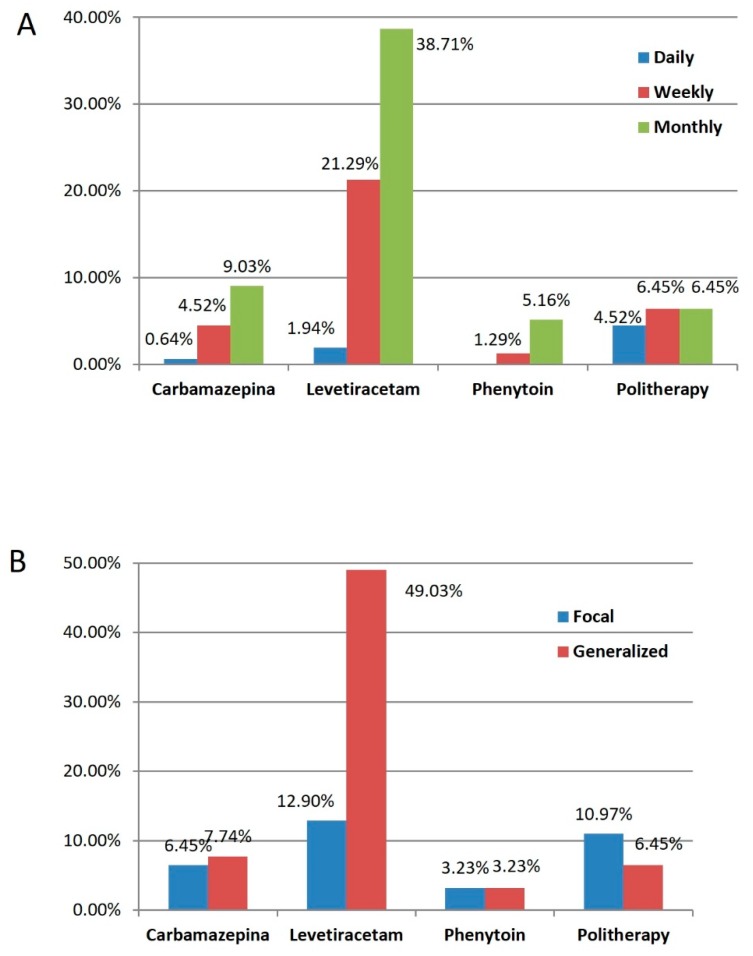
Graph illustrating the preoperative Anti-Epileptic Drugs (AESs) stratified by seizure frequency (**A**) and by onset seizure type (**B**).

Demographic, clinical, histological, molecular, and radiological data of the 155 DLGG patients included in the study are summarized in Table 1 and Table 2. Seizure features are plotted in Figure 1 according to the preoperative Anti-Epileptic Drugs (AEDs). Overall, the median duration between seizure onset and surgery was 6 months (range 4–20 months). The preoperative MRI evidenced in all cases the absence of enhancement on T1-weighted post contrast MRI sequences. The median preoperative tumor volume, computed on T2-weighted MR images, was 48 cm^3^ (range 6–144 cm^3^). The median preoperative ΔT2T1 MRI value was 12 cm^3^ (range 0–55 cm^3^). The median EOR was 88% (range 38%–100%) and the median postoperative residual tumor was 10 cm^3^ (range 0–44). According to the 2016 WHO classification of the brain tumors, based on morphology and molecular alterations, 44 oligodendroglioma, IDH-mutant, and 1p/19q codeleted, 93 diffuse astrocytoma, IDH-mutant, and 18 diffuse astrocytoma, IDH-wild type, were identified.

### 2.2. Postoperative Seizure Outcome Analysis

**Figure 2:**
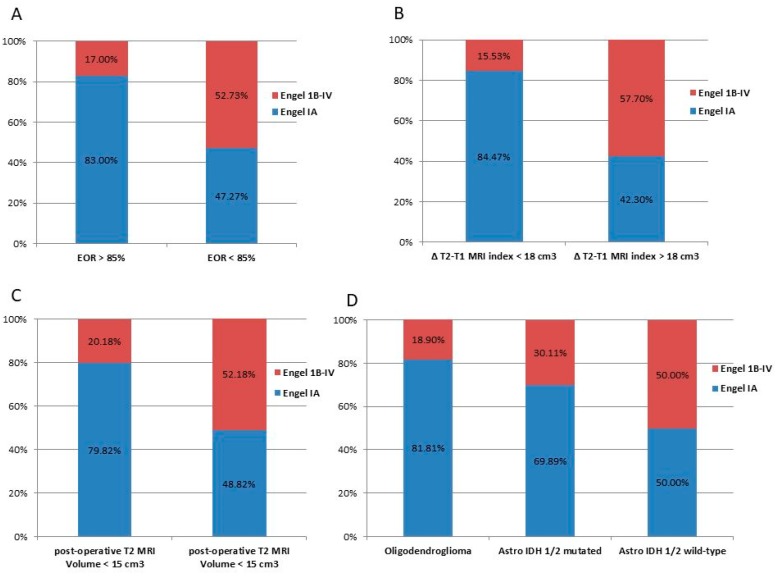
Graph illustrating 18-months postoperative seizure outcome stratified by the significant variables at univariate analysis. Blue bars indicate patients in Engel Class IA; yellow bars indicate those in Engel Class IB or above. Individual bar totals are the total number of patients with postoperative seizures within each category. (**A**) Distribution of patients stratified by the EOR; (**B**) Distribution of patients stratified by the preoperative tumor growing pattern expressed by the ΔT2T1 MRI index; (**C**) distribution of patients stratified by the residual tumor computed on T2 weighted images; (**D**) distribution of patients stratified by the molecular class according to the 2016 WHO classification.

Postoperative seizure outcome was proportionally similar at the four analyzed time points (4, 8, 12, and 18-month follow-up). By considering the entire cohort of 155 cases, postoperative seizure control at 18 months was as follows: 110 patients (70.97%) were classified as Engel Class IA (completely seizure free), 16 patients (10.32%) as Engel Class IB-ID, 23 patients (14.84%) as Engel Class II-III, and six patients (3.87%) as Engel Class IV. The distribution, according to different postoperative seizure outcome (Engel Class IA vs. IB-IV), of EOR, residual tumor, molecular class, and preoperative Δ*T2T1 MRI* index, and intraoperative protocol are represented in Figure 2.

Patients of Engel Class IB-IV required changes in AEDs therapy to optimize seizure control after surgery. As shown by the 18-month postoperative follow-up evaluation, these therapeutic changes, however, failed to produce complete seizure freedom, and no other patients achieved Engel Class IA.

AEDs changes was usually done for patients belonging to Engel Class IA and IB. For all the others, we proceeded with changings and/or optimization of the pharmacological treatments (i.e., add on therapy, a new AED as monotherapy, increased posology). Specifically, further surgery, radiotherapy or chemotherapy were not considered, in patients not seizure free after surgery, based on the fact that all cases showed no signs of tumor progression within the follow-up considered.

At univariate analysis the following parameters were associated with postoperative seizure outcome: frequency of preoperative seizures; seizure-onset features; preoperative ΔT2T1 MRI index; molecular class; EOR; AEDs in mono- or poly-therapy; and postoperative residual tumor computed on T2-weighted images. 

In a multivariate analysis model which considered all these variables, only age (*p* = 0.014), ΔT2T1 MRI index (*p* = 0.016), and EOR (*p* = 0.030) were shown to be independent predictors of outcome (Table 3).

### 2.3. ROC Analysis 

**Figure 3:**
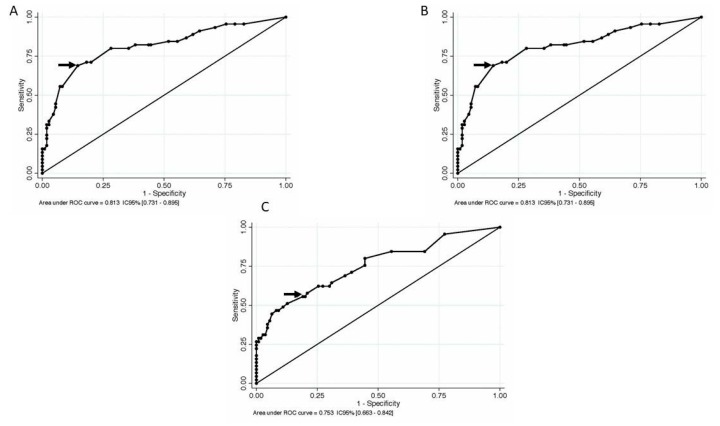
A receiver operating characteristic (ROC) curve for EOR, preoperative ΔT2T1 MRI index, and postoperative residua tumor on T2-weighted images, to predict seizures relapse after surgery. The optimal diagnostic point is the one with maximal sensitivity and specificity. It is the point closest to the top left corner of the graph, indicated by the arrow. (**A**) The optimal threshold corresponded to an EOR of 85%, which was the point with the highest sensitivity (0.764) and specificity (0.644), with a resulting area under the curve of 0.783 (CI 95% 0.700–0.865) and a predictive accuracy of 72.90%; (**B**) for the preoperative ΔT2T1 MRI index, the threshold of 18 cm^3^ corresponded to the point with the highest sensitivity (0.689) and specificity (0.855), with a resulting area under the curve of 0.813 (CI 95% 0.731–0.895) and a predictive accuracy of 82.65%; (**C**) regarding the residual tumor, the optimal threshold corresponded to 15 cm^3^, which was the point with the highest sensitivity (0.556) and specificity (0.809), with a resulting area under the curve of 0.753 (CI 95% 0.663–0.842) and a predictive accuracy of 73.55%.

In order to determine statistical clinical useful postoperative seizure outcome cutoff predictive values for EOR, preoperative ΔT2T1 MRI index and residual tumor, a receiver operating characteristic (ROC) curve was computed based on Engel class, using a binary outcome (Engel Class IA versus IB-IV) (Figure 3).

The optimal threshold corresponded to an EOR of 85%, which was the point with the highest sensitivity (0.764) and specificity (0.644), with a resulting area under the curve of 0.783 (CI 95% 0.700–0.865) and a predictive accuracy of 72.90%.

Regarding the residual tumor, the optimal threshold corresponded to 15 cm^3^, which was the point with the highest sensitivity (0.556) and specificity (0.809), with a resulting area under the curve of 0.753 (CI 95% 0.663–0.842) and a predictive accuracy of 73.55%.

For the preoperative ΔT2T1 MRI index, the threshold of 18 cm^3^ corresponded to the point with the highest sensitivity (0.689) and specificity (0.855), with a resulting area under the curve of 0.813 (CI 95% 0.731–0.895) and a predictive accuracy of 82.65%.

**Figure 4:**
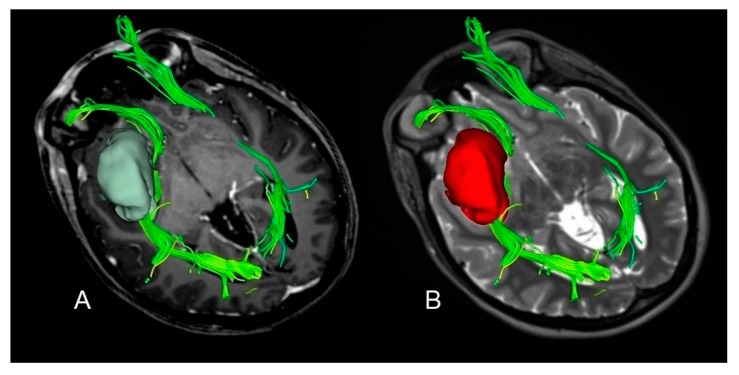
A case of insular diffuse low grade gliomas (DLGG) with a regular shape, determining similar tumor volume in both post-contrast T1-weighted MRI and T2-weighted MRI sequences and displacing the Fronto-Occipital Longitudinal Fasciculus. The preoperative tumor volume computed on post-contrast T1-weighted magnetic resonance imaging (MRI) was 32 cm^3^ (axial slices **A**). The preoperative tumor volume computed on T2-weighted MRI was 34 cm^3^ (axial slices **B**). The preoperative ΔT2T1 MRI index was 2 cm^3^, showing the prevalence of the proliferative tumor growing pattern. The patient was in Engel Class IA 18 months after surgery.

**Figure 5:**
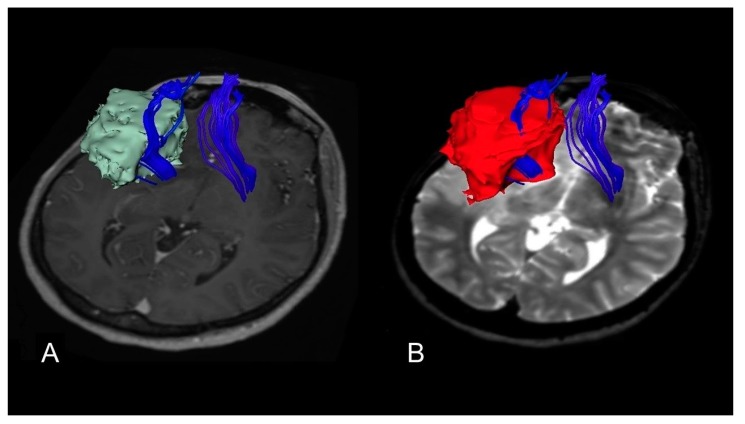
A case of insular DLGG infiltrating the Cortico-Spinal tract. The preoperative tumor volume computed on post-contrast T1-weighted magnetic resonance imaging (MRI) was 58 cm^3^ (axial slices (**A**)). The preoperative tumor volume computed on T2-weighted MRI was 83 cm^3^ (axial slices (**B**)). The preoperative ΔT2T1 MRI index was 25 cm^3^. The prevalence of the diffusive and infiltrative growth generates the tumor diffusion along the white matter, resulting in a complex shape with digitations more visible on T2-weighted images. The patient was in Engel Class IB 18 months after surgery.

Based on the preoperative ΔT2T1 MRI index an example of proliferative and diffusive DLGG are shown in Figure 4 and Figure 5.

## 3. Discussion

In this retrospective study, which included 155 adult patients DLGG with preoperative drug-resistant tumor related epilepsy, postoperative seizure outcome was analyzed stratifying the results by tumor volumetric data and molecular markers. 

This study showed the following:

(1) 70.97% of epileptic DLGG patients were in Engel Class IA 18 months after surgery;
(2) Improved postoperative seizure outcome can be expected for EOR ≥ 85%, residual tumor ≤ 15 cm^3^, and preoperative ΔT2T1 MRI index ≤ 18 cm^3^.
(3) Tumor infiltration index, expressed by ΔT2T1 MRI index, represents a quantitative evaluation of the diffusive and infiltrative tumor component as predictor of postoperative seizure outcome.
(4) IDH1/2 mutation may represent the prevalent epileptogenic mechanism in presence of higher ΔT2T1 MRI index and consequent lower EOR.

### 3.1. The Role of EOR 

The treatment paradigm of DLGGs is based on the principle of the onco-functional balance, which implies maximization of EOR with preservation of quality of life.

The role of EOR as the strongest predictors of postoperative seizure long-term outcome (Table 4) has recently been demonstrated in a limited number of retrospective studies [8,15,16,17,19,20,21,22,23]. Only two investigations examined which value of EOR corresponded to the threshold above which seizure control, defined as Engel Class level, was optimal [16,17].

Xu et al. showed the existence of an EOR threshold for long-term seizure freedom corresponding to an EOR > 80% [17].

In a subsequent larger multi-center investigation, methodologically well-designed, Still et al. demonstrated that postoperative seizure control was more likely when EOR was ≥91% and/or when residual tumor volume was ≤ 19 cc in supratentorial DLGG patients [16].

The results reported in these studies with a postoperative follow-up of 6 months were also confirmed in our study, however, with a follow-up period of 18 months. Our data showed an optimum EOR threshold of ≥ 85% and a residual tumor threshold of 15 cm^3^ to be associated with a higher likelihood of postoperative seizure control at one year. The epileptogenic focus and the tumor are not always overlapped. Hippocampectomy and corticectomy combined with lesionectomy in patients with DLGG and intractable preoperative tumor related epilepsy have been shown to improve seizure control [24]. The use of intraoperative electrocorticography (ECoG) to identify epileptogenic areas, guide surgical strategy, and improve postoperative seizure control in patients with LGGs still remains inconclusive, mainly due to its low accuracy to detect distant epileptogenic focus and to follow the spreading of epileptic activity [25]. Future intraoperative prospective studies are required to combine the intraoperative use of direct electrical stimulation (DES) and ECoG in order to optimize the DLGG surgery not only in terms of EOR achievable but also for the postoperative seizure outcome. Intraoperative integration between DES and ECoG may allow a supratotal resection, beyond the radiological margins of the tumor, when functionally possible.

### 3.2. The Tumor Growth Pattern Influences the Postoperative Seizure Outcome

Changes in peritumoral tissue are involved in in the pathogenesis of tumor-related epilepsy [1,2,6,8,15,26,27].

Furthermore, during the sprouting of tumor cells in normal tissue, glioma cells release a high level of glutamate into the extracellular space. As a consequence, an imbalance between inhibitory and excitatory mechanisms is induced, generating neuron cell death, and promoting the migration of tumor cells [6,27].

As previously described, the preoperative ΔT2T1 MRI index provides an imaging estimate of tumor growing pattern prevalence and the EOR achievable [12,15]. 

Kinetic analysis in low grade gliomas highlighted that tumor growth results from two main mechanisms: proliferation and diffusion [28,29].

The prevalence of proliferation growth leads to a bulky tumor with a regular shape, determining similar tumor volume in both post-contrast T1-weighted MRI and T2-weighted preoperative MRI sequences; whereas the prevalence of the diffusive growth generates the tumor infiltration along the white matter, resulting in a complex shape with digitations more visible on T2-weighted images and less amenable of an extensive tumor resection [28,29].

Higher level of preoperative ΔT2T1 MRI index thus represents the prevalence of the diffusive growing mechanism.

In this study, we confirmed the role played by the tumor growth pattern (proliferative versus diffusive), expressed by ΔT2T1 MRI index on postoperative seizure outcome [15].

As a new feature, we provided a predictive cut-off value by the receiver operating characteristic (ROC) analysis. 

According to ROC analysis, the optimum ΔT2T1 MRI index threshold ≤ 18 cm^3^ was associated with a higher likelihood of long-term seizure control. Otherwise patients with a ΔT2T1 MRI index >18 cm^3^ had a higher likelihood of postoperative seizure persistence at 18 months. 

Assuming that the preoperative ΔT2T1 MRI index higher than 18 cm^3^ reflects the prevalence of the diffusive and infiltrative tumor component, it could constitute an indirect imaging evaluation of changes in peritumoral tissue induced by tumor growth.

When the diffusive mechanism is predominant, tumor infiltrates the functional area limiting the resection, thus ΔT2T1 MRI index may provide a potentially estimation of the epileptic network development, allowing the preoperative detection of patients at greater risk of postoperative seizure persistence.

### 3.3. It is a Matter of Interaction between EOR and Tumor Growth Pattern

The simultaneous role played by the EOR and the ΔT2T1 MRI index in postoperative seizure outcome could reflect the relationship between the extent of resection achievable and the tumor growing pattern. The ΔT2T1 MRI index is the prognostic preoperative index of EOR itself, while the EOR is inversely related to the tumor growth pattern, as demonstrated in our previous study [12,15].

We can thus assume that the less infiltrative the tumor growth pattern is, the better the chances of greater EOR are and, consequently, the better the postoperative seizure control is.

Regarding the molecular assessment, only a few investigations have focused on the role played by the molecular biomarkers on postoperative seizure outcome in DLGG patients, reaching divergent results [21,23].

In a recent investigation, Neal et al. found a strong relationship between the higher IDH1-R132H rates and a severe postoperative seizure outcome, although the contribution to tumor related epilepsy by IDH 1/2mutation is not clear [21].

In contrast, Zhong et al. reported no significant difference in IDH status and seizure outcomes in 222 patients with WHO grade II gliomas [23].

In our study, when comparing seizure outcomes in patients with oligodendrogliomas and with astrocytomas (based on the 2016 WHO update), the majority of patients with oligodendrogliomas were Engel Class IA in comparison with patients with astrocytoma IDH1/2 mutated or astrocytoma IDH1/2 wild type (81.82%, 68.89%, and 50%, respectively; *p* = 0.001). It is likely that LGGs, which show less infiltrative growth, as oligodendrogliomas, are less prone to modify the tumor microenvironment, comparing to infiltrative lesions. Thus, electrical signaling of peritumoral tissues may be less impaired in oligodendrogliomas [20].

As a further confirmation regarding the role of EOR on seizure outcome, the Cox analysis revealed that the tumor molecular class did not result as an independent predictor at multivariate analysis, suggesting that EOR and the peritumoral infiltrative component are more important in predicting the outcome of epilepsy. Considering that the ΔT2T1 MRI index represents the infiltrative component of the tumor, it could be an indirect index of changes in peritumoral tissue induced by tumor growth and infiltration [15,26,27]. This index may be considered as a measure of persistent epileptogenic process after surgery. It is interesting to note that patients with prevalence of infiltrative tumor growing pattern, which means ΔT2T1 MRI index higher than 18 cm^3^, had a worse seizure outcome (66% of patient in Engel Class IB-IV, Figure 1B) and expressed the IDH1-2 mutation in more than 90% of cases. Huberfeld et al., in 2016, explained the relationship between epilepsy in glioma and IDH1/2 expression. 

The epileptic discharge and tumor proliferation can be traced back to an imbalance in glutamate transporters determining an increase in concentrations of extracellular glutamate [6]. The presence of IDH mutated cells can explain seizure persistence in patients with a reduced EOR and high ΔT2T1 MRI index in the IDH-mutated tumors.

This hypothesis may explain the negative epileptic outcome in patient with higher ΔT2T1 MRI index. Indeed, at multivariate analysis, the ΔT2T1 MRI index (*p* = 0.016) resulted as a stronger predictor of postoperative outcome in comparison with EOR (*p* = 0.030), underlying the importance of the infiltrative tumor component, which is generally not removed because it is functional, in seizure persistence. The availability of predictive factors for postoperative seizure outcome could provide a useful tool to guide therapeutic antiepileptic strategy after surgery, avoiding pharmacological overtreatment improving the patients’ quality of life.

### 3.4. Limitation and Future Directions

There are several limitations of our study. The most important one is based on the retrospective nature of the investigation

The peritumoral cortex may contain epileptogenic foci, which may directly affect postoperative seizure control [8,24,30]. Future studies are thus required to better investigate the correlation between supra-total resection, when functionally achievable, and seizure outcome.

Although in the present study gliomas were classified accordingly to the currently used 2016 WHO classification of the brain tumors, it is desirable in the future to molecularly characterize diffuse astrocytic gliomas, IDH-wildtype, in order to recognize those that can indeed be classified as diffuse astrocytic glioma, IDH-wildtype, with molecular features of glioblastoma, WHO grade IV [31,32]. Additional information deriving from next generation sequencing analysis would help stratify postoperative seizure outcome in DLGGs, using markers with known pathophysiological roles in epilepsy such as glutamate metabolism/clearance [5].

In the pattern of results reported, the cognitive statuses of the patients were not considered. The impact of tumor related epilepsy on the pre-surgical neuropsychological examination could prove to be useful to better assess the effects of tumor growth itself and the influence of tumor related epilepsy or medication on the cognitive status of the patients. It has been shown that DLGG patients can present emotional and personality changes in their post-surgery examination [33]. An important aspect would be analyzing the effects of tumor related epilepsy on the post-surgery quality of life of patients in terms of emotional processing and personality. 

In closing, considering the lack of standardized protocol for tumor related epilepsy management, both before and after surgery, it should be important to plan a multidisciplinary approach considering the complex therapeutic profile of DLGGs patients [34]. In detail, a preoperative study as for epilepsy surgery with prolong Video-EEG recordings for patients with tumor related epilepsy characterized by complex semiology not directly associated with tumor location, could be useful in future studies to assess the spreading of epileptic discharges and plan the function possibility of resection and the intra-operative position of the strips.

A tailored AEDs treatment should be adopted for each patient, considering that changes in seizure type or worsening in Engel Class could be related to tumor progression. There are numerous points of interest to be noted: homogeneous data collection in a monoinstutional case-series; strict definition of postoperative seizure control as Engel Class A patients; postoperative seizure follow-up at 18 months after surgery concomitant with control MRI, to rule out cases with tumor progression; integration of tumor volumetric data and molecular data, according to 2016 WHO classification, to stratify postoperative seizure outcome; ROC analysis to determine EOR, residual tumor volume, and ΔT2T1 MRI index corresponding to optimal seizure outcome.

## 4. Materials and Methods 

### 4.1. Study Population 

A total of 155 adult patients with tumor related epilepsy underwent surgery at our institution for primary DLGG (January 2007–May 2018). 

Seizures were the onset symptom and all patients developed seizure not fully controlled with medical treatment before surgery. At least two AEDs were given in successive monotherapies or together in politherapy, resulting in a drug-resistant epilepsy, according to the International League Against Epilepsy (ILAE) definition [35].

The 2017 ILAE classification was applied to classify the type of seizures [36].

Patients were enrolled according to the following criteria: age ≥ 18 years; preoperative MRI suggestive of supratentorial low grade glioma; no previous surgery, chemo- or radio-therapy; at least 18 months of follow up, with concomitant MRI control, to rule out cases with tumor progression. Objective evaluation of EOR preoperatively and postoperatively on MRI images in DICOM format based on T2-weighted MRI sequences; revision of histopathological specimens by using the new 2016 WHO Classification; diagnosis of drug-resistant tumor related epilepsy, according to the ILAE definition [37].

Patients were evaluated preoperatively, at discharge and, during the follow up, every 6 months. Engel Class at 18-month follow-up was used to compute predictors of postoperative seizure outcome [36].

No patient underwent adjuvant therapy with radiotherapy or chemotherapy during the period of postoperative follow-up.

The local Ethics Committee, Comitato Etico Unico Regionale del Friuli Venezia Giulia, approved this investigation (protocol N.0036567/P/GEN/EGAS, ID study 2540). Considering that the study was retrospective, written consent to participate in the study was not applicable. Written informed consent was obtained for surgery.

### 4.2. Surgical Procedure 

All patients were surgically treated with the aim of the intraoperative brain mapping technique both at cortical and subcortical level [38].

The awake surgery protocol was selected following the standard protocol previously described [39].

### 4.3. Volumetric Analysis

Tumor volume data were obtained by analyzing structural imaging data routinely acquired during pre-surgery and post-surgical investigations in axial 3D T2-weighted and 3D post-contrast T1-weighted MRI slices. All tumor segmentations were realized by using the OSIRIX software tool (GNU LESSER, General Public License, Geneva, Switzerland) [40,41]. Specifically, the tumor growing pattern, expressed by MRI ΔT2T1 index, and EOR were computed as previously described [12,15].

Briefly, the tumor growing pattern and the EOR were assessed as listed: (1) preoperative tumor volume segmented on axial T2-weighted MRI images-preoperative tumor volume segmented on axial T1-weighted images. (2) (preoperative tumor volume-postoperative tumor volume)/(preoperative tumor volume) in axial T2-weighted MRI axial images.

### 4.4. Histological and Molecular Analysis 

Histological and molecular data were retrospectively analyzed according to the 2016 WHO classification [42].

Immunohistochemistry (IHC) for Ki67 and IDH1R132H, fluorescence in situ hybridization (FISH) to evaluate 1p/19q codeletion and analysis of the genetic status of O6-methylguanine-DNA-methyltransferase (MGMT) promoter and isocitrate dehydrogenase (IDH1/2) genes were performed as previously described. Gliomas were classified as methylated when the average percentage of methylation of CpG islands was ≥ 8% [43].

### 4.5. Statistical Analysis

Categorical variables were reported as percentages, continuous variables were reported as mean ± standard deviation or median and range as appropriate, according with the data distribution. Normality of the continuous variables was tested using the Shapiro–Wilk test, the *t*-test or Mann–Whitney U-test as appropriate, was used to compare continuous variables between groups. For the outcome analysis, Engel classification was dichotomized as Class IA versus Class IB-IV (patients were either completely seizure free or not completely seizure free). In univariate analysis, the variables considered as possible prognostic factors were age, sex, preoperative tumor volume, tumor histological subtype, molecular markers, tumor side, preoperative seizures feature, seizure onset characteristics and frequency, time between seizure onset and surgery, intraoperative protocol used, EOR, residual tumor volume, and preoperative ΔT2T1 MRI index. 

Multivariate stepwise backward analyses included all variables significant at *p* = 0.05 in univariate analysis. The results were presented as odds ratios and 95% confidence intervals. 

For EOR, preoperative ΔT2T1 MRI index and residual tumor threshold, the cut-off values able to discriminate, with high sensitivity and specificity, the postoperative seizure control, was determined by De Long’s nonparametric receiver operating characteristic (ROC) analysis with exact binomial estimation of confidence intervals (CI) of the area under the curve (AUC).

All analyses were conducted using Stata/SE (version 14.0 Stata Corp.) for Mac. All two-tailed statistical significance levels were set at *p* < 0.05.

## 5. Conclusions

The association between a worse seizure outcome and the prevalence of an infiltrating tumor growing pattern expressed by high ΔT2T1 MRI index reinforces evidence of common mechanisms underlying both epileptogenesis and tumor growth.

The individual evaluation of the tumor growing pattern and the estimation of the EOR may thus represent a helpful tool in the early identification of patients with an increased risk of seizure persistence after surgery.

Tumor volumetric information could be useful when deciding and discussing prognosis and potential postoperative seizure outcomes to better handle the entire management, starting from pre- to post surgery.

Considering the role of IDH 1/2 mutation in both tumor growing and epileptogenesis, and considering that up to 80% of DLGGs carried out this mutation [44], potential future goals could be represented in the development of anti-epileptic drugs targeting the underlying biochemical pathology related to the mutation of IDH 1/2.

## Figures and Tables

**Table 1:** Baseline characteristics of the study population. Characteristics of the study population are described using means ± s.d. (standard deviation) or median and range for continuous variables, number of cases with relative percentages reported in parentheses for categorical variables. Abbreviations: ΔT2T1 MRI index, volumetric difference between preoperative tumor volumes on T2 and T1 weighted MRI images; EOR, extent of surgical resection.

Parameters	Value (N and %, Mean ± s.d. or Median and Range)
No. of patients	155
Sex	
Female	59 (38.06%)
Male	96 (61.94%)
Age (years)	37 18-73)
Tumor side	
Left	88 (56.77%)
Right	67 (43.23%)
Tumor site	
Frontal	50 (32.26%)
Parietal	13 (8.39%)
Temporal	24 (15.48%)
Insular	65 (41.94%)
Occipital	3 (1.93%)
Preoperative tumor volume computed on T2-weighted MRI images, cm^3^	48 (6–144)
Preoperative ΔT2T1 MRI index, cm^3^	12 (0-55)
Preoperative ΔVT2T1 MRI index	
<18 cm^3^	88 (56.67%)
≥18 cm^3^	67 (43.23%)
EOR%	88 (38–100)
EOR%	
100	30 (19.36%)
99–90	43 (27.74%)
70–89	45 (29.03%)
≤69	37 (23.87%)
Postoperative residual tumor volume computed on T2-weighted MRI images, cm^3^	10 (0–44)
Molecular class	
Oligodendroglioma IDH1/2 mutated 1p-19q codeleted	44 (28.39%)
Astrocytoma IDH 1/2 mutated 1p-19q non codeleted	93 (60.00%)
Astrocytoma IDH 1/2 wild type	18 (11.61%)
MGMT Methylation status yes vs. no	136 vs. 19 (87.74% vs. 12.26%)
Intraoperative protocol	
Awake surgery	113 (72.90%)
General anesthesia	42 (27.10%)
Time between seizure onset and surgery	6 months (range 4–20 months)

**Table 2:** Seizure characteristics. Abbreviations: AEDs, anti-epileptic drugs.

Parameter	N (%)
**Onset Seizure Features**	
Focal	52 (33.55%)
Motor	18
Non motor sensory	17
Non motor emotional	2
Non motor cognitive	11
Non motor autonomic	4
Generalized	103 (66.45%)
Motor	76
Focal to bilateral	14
Absence	9
Non motor cognitive	2
Non motor emotional	1
Non motor sensory	1
Seizure Frequency	
Monthly	92 (59.35%)
Weekly	52 (33.55%)
Daily	11 (7.10%)
**Duration**	
<1 year	133 (85.81%)
>1 year	22 (14.19%)
Preoperative AEDs	
Levetiracetam	96 (61.94%)
Polytherapy	27 (17.42%)
Carbamazepine	22 (14.19%)
Phenytoin	10 (6.45%)
Postoperative Engel Class	
IA	110 (70.97%)
IB, IC, ID	16 (10.32%)
II, III	23 (14.84%)
IV	6 (3.87%)
Postoperative AEDs	
Levetiracetam	105 (67.74%)
Polytherapy	31 (20.00%)
Oxcarbamazepina	6 (3.86%)
Carbamazepine	5 (3.23%)
Valproic Acid	5 (3.23%)
Lacosamide	3 (1.94%)

**Table 3:** Predictors of 18 months postoperative seizure control (Engel IA) on univariate and multivariate analyses. Boldfacing values represent statistically significant results (*p* < 0.05).

Variable	Univariate Analysis			Multivariate Analysis		
	Odds Ratio	95% CI	*p*-Value	Odds Ratio	95% CI	*p*-Value
Age (yrs)	1.042	1.010–1.074	**0.009**	1.056	1.010–1.103	**0.014**
Sex						
Male	1					
Female	1.456	0.718–2.950	0.297			
Tumor side						
Left	1					
Right	1.044	0.290–3.758	0.947			
Tumor Site						
Pre-central	1					
Retro-central	0.755	0.207–2.752	0.671			
Temporal	0.497	0.159–1.552	0.229			
Insular	0.723	0.328–1.591	0.421			
Onset seizure features						
Generalized	1					
Focal	1.057	0.324–2.267	0.013			
Seizure frequency						
Monthly	1					
Weekly	1.457	0.690–3.076	0.323			
Daily	2.500	0.697–8.966	0.160			
Duration						
<1 yr	1					
>1 yr	0.857	0.324–2.267	0.756			
Preoperative tumor volume computed on T2-weighted images, cm^3^	1.116	1.069–1.185	<0.0001			
ΔT2T1 MRI index	1.156	1.066–1.195	<0.0001	1.077	1.102–1.134	0.016
Molecular Class						
Astrocytoma IDH1/2 mutated 1p-19q non codeleted	1					
Astrocytoma IDH1/2 wild type	0.430	0.154–1.200	0.107			
Oligodendroglioma IDH1/2 mutated 1p-19q codeleted	0.222	0.669–0.747	0.014			
MGMT Methylation yes vs. no	2.382	0.658–8.619	0.186			
% EOR Continuous variable	0–929	0.903–0.955	<0.0001	0.957	0.920–0.995	0.030
Postoperative residual tumor volume computed on T2 weighted MRI images, cm^3^	1.057	0.324–2.267	0.001			

**Table 4:** Postoperative tumor related epilepsy outcome in DLGGs: Literature review stratified by EOR and 2016 WHO molecular features.

Authors	N of Cases	Age at Surgery (years)	Location	Histology	Preoperative Tumor Volume cm^3^	EOR	Preoperative Seizures	Postoperative Seizures (Engel Class I Outcome)	IDH1/2 Mutation	1p/19q Codeletion	MGMT Methylation	P53+
Neal A et Al. 2018 [21]	70 HGG and 30 LGG	50.2 ± 17.5	Frontal 48; occipital 1; parietal 11; temporal 26	70 HGG 20 A 10 O/OA	NA	15 PR 42 ST 37 GTR 6 unknown	52 cases (52%)	58 cases (58%)	35 cases (35%)	NA	NA	NA
Still M.E.H. et al. 2018 [16]	346 LGG	35.0	Frontal 192, temporal 70, insular 41, parietal 27, other 16	48 A 298 O	NA	100% 50 cases; 90%–99% 92 cases; 50%–89% 134 cases; <50% 70 cases	346 cases (100%)	227 cases (65.60%)	19 (21 cases tested) (90.47%)	65 (206 cases tested) (31.55%)	NA	NA
Xu DS et al. 2018 [17]	128 LGG	40.8	Frontal 74, parietal 34, temporal 45, occipital 8, insular 17, deep 6	18 A 86 O 24 OA	57,5	90%–99% 64 cases; 80%–89% 11 cases	128 cases (100%)	105 cases (82.03%)	NA	25 cases (19.53%)	NA	NA
Chen H et al. 2017 [19]	712 GLIOMA	54 (60.7–53.4)	Temporal 191 non temporal 521	77 WHO II, 128 WHO III, 507 WHO IV	NA	NA	276 cases (38.76%)	NA	177 cases (16.43%)	644 cases (90.44%)	NA	NA
Zhong Z. et al. 2015 [23]	311 LGG	38	NA	140 A 140 OA 31 O	NA	NA	183 cases (58.84%)	211 cases (67.84%)	257 cases (82.63%)	NA	NA	NA
Yang Y. et al. 2015 [22]	6 LGG 106 HGG	34 (39.8–42.2)	88 frontal; 74 temporal; 45 parietal; 11 occipital; 17 insular	64 WHO II 58 WHO III 48 WHO IV	4.7 cm (5.6–6.4 cm)	NA	74 cases (42.3%)	NA	41 WHO II cases (64.0%); 33 WHO III cases (56.8%); 10 WHO IV cases (20.8%)	NA	NA	24 WHO II cases (37.5%); 28 WHO III cases (48.2%); 25 WHO IV cases (52.0%)
Ius et al. 2014 [15]	52 LGG	38.73	Insula; left 36, right 16	32 A 11 OA 9 O	75.42	87% >90% 21 cases 70–89% 23 cases <70% 8 cases	NA	35 cases (67.30%)	NA	NA	NA	NA
Mulligan L. et al. 2014 [20]	62 LGG	NA	NA	62 O	4 groups: 45 mm 46 mm 56 mm 37.5 mm	NA	48 cases (77.41%)	NA	48 cases (77.41%)	39 cases (62.90%)	NA	24 cases (38.70%)
Liubinas SV et al. 2014 [8]	30 LGG	35.4 years	NA	22 A 6 OA 1 mixed OA and protoplasmic astrocytoma 1 O	4 groups: 45 mm, 46 mm, 56 mm, 37.5 mm	NA	23 cases (76.66%)	NA	17 cases (56.66%	NA	NA	NA
Pallud J et al. 2014 [7]	1509 LGG	<30 yrs = 390 cases, 30–45 yrs = 726 cases	NA	327 A 781 OA 280 mixed glioma 121 missing	NA	<100 cm^3^ 808 cases (53.54%), >100 cm^3^ 346 cases (22.92%), missing cases 355 (23,54)	NA	NA	NA	NA	NA	NA

A = astrocytoma; EOR = extent of resection: GTR = gross-total resection; HGG = high grade glioma; LGG = low grade glioma; NA = not applicable; PR = partial resection: OA = oligoastrocitoma; O = oligodendroglioma; STR = subtotal resection.
